# Bibliometric and visual analysis of omega-3 polyunsaturated fatty acids in cancer research (2000–2025)

**DOI:** 10.3389/fnut.2026.1818038

**Published:** 2026-07-08

**Authors:** Cuicui Gao, Yanyan Li, Hui Su, Xiao Yu, Xiangqian Lu, Xin Liang, Feifei Tao, Xiaomeng Duan

**Affiliations:** 1 Department of Clinical Nutrition, Liaocheng People’s Hospital, Liaocheng, China; 2 Department of Oncology, Liaocheng People’s Hospital, Liaocheng, China; 3 Department of Radiation Oncology, Liaocheng People’s Hospital, Liaocheng, China

**Keywords:** applications, bibliometrics, cancer, epidemiology, inflammation, omega-3 PUFAs, therapeutic

## Abstract

**Objective:**

Omega-3 polyunsaturated fatty acids (PUFAs) have received considerable attention as potential adjunctive agents in antitumor therapy. However, systematic bibliometric analyses of this research area remain limited. This study aims to systematically examine research trends, knowledge hotspots, and emerging themes related to omega-3 PUFAs in oncology from 2000 to 2025, with the goal of providing valuable insights for future investigations.

**Methods:**

Records related to omega-3 PUFAs and oncology published between 2000 and 2025 were retrieved from the Web of Science Core Collection and Scopus. Bibliometric mapping was performed using R/bibliometrix, VOSviewer, and CiteSpace. In addition, clinical trial records from PubMed were screened to summarize clinical progress in this field.

**Results:**

A total of 2,917 publications from the Web of Science and 7,380 publications from Scopus were analyzed. From 2000 to 2025, the annual number of publications in this field increased gradually, then reached a plateau, followed by a slight decline. The United States was the leading contributor in terms of publication output (*n* = 819), followed by China (*n* = 391) and Italy (*n* = 171). The United States also demonstrated a broader international collaboration network. Nutrients published the largest number of articles (*n* = 98, IF = 5.0), whereas the American Journal of Clinical Nutrition received the highest number of citations (*n* = 5,299, IF = 6.9). Major research themes included “inflammation,” “breast cancer,” “risk,” “colorectal cancer,” and “expression.” Antitumor mechanisms represented the dominant research focus. Another major theme involved associations among dietary omega-3 PUFAs intake, biomarker status, and cancer risk or progression. Furthermore, the application of omega-3 PUFAs in clinical antitumor therapy has emerged as a prominent research trend. Clinical trials in this field have primarily focused on antitumor applications and have shown a tendency toward combined treatment strategies.

**Conclusion:**

Research on omega-3 PUFAs in oncology is progressively shifting from mechanistic exploration toward clinically oriented and translational applications. Emerging trends emphasize the integration of molecular mechanisms, objective exposure biomarkers, and clinical intervention strategies within a precision-oriented nutritional oncology framework. Future studies should focus on standardized exposure assessment, dose–response modeling, patient stratification, and clinically meaningful endpoints to better define target populations, optimal formulations and dosages, and appropriate intervention windows.

## 1 Introduction

Cancer represents a substantial and pervasive global public health threat, markedly reducing patient survival and quality of life while imposing a considerable socioeconomic burden. According to the latest statistics released by the World Health Organization (WHO) and the International Agency for Research on Cancer (IARC), approximately 19.965 million new cases of malignant tumors were diagnosed worldwide in 2022, resulting in 9.737 million deaths (1). By 2050, the number of newly diagnosed cancer cases worldwide is projected to exceed 35 million (1). Current major cancer treatments include surgery, chemotherapy, radiotherapy, targeted therapy, and immunotherapy. Despite significant advances in oncology, the effectiveness of existing therapies remains limited. For example, chemotherapy and targeted therapies often lead to drug resistance, which is a major cause of treatment failure and tumor recurrence (2). Moreover, anticancer drugs are frequently associated with severe toxicities, including fatigue, nausea and vomiting, anorexia, neurotoxicity, and thrombosis. These adverse reactions substantially impair patients’ quality of life (3). Therefore, cancer prevention and treatment continue to face considerable challenges. Effective adjunctive strategies are urgently needed to complement conventional therapies, reduce adverse reactions, and improve therapeutic outcomes. In this context, nutritional interventions with anti-inflammatory and immunomodulatory properties have attracted increasing attention as potential adjunctive strategies in oncology. Among these interventions, omega-3 polyunsaturated fatty acids (omega-3 PUFAs) are of particular interest because of their broad biological activities and potential relevance to cancer prevention and treatment (4, 5).

Omega-3 PUFAs are a group of unsaturated fatty acids characterized by the presence of the first double bond at the third carbon atom from the methyl (ω) end. In nutritional and oncology-related studies, alpha-linolenic acid (ALA), eicosapentaenoic acid (EPA), and docosahexaenoic acid (DHA) are the most commonly investigated omega-3 PUFAs because of their major dietary sources, biological relevance, and relatively well-established roles in human health (6, 7). ALA is an essential fatty acid that cannot be synthesized by the human body and must therefore be obtained through dietary intake. It is primarily derived from nuts and certain plant oils, including chia seed oil, walnut oil, flaxseed oil, and hemp seed oil. EPA and DHA, which have longer carbon chains and a higher degree of unsaturation, are mainly found in fatty fish, seafood, and algal oils. Their biological efficacy is substantially greater than that of ALA (8). Although ALA can be converted into EPA and DHA in the body through a series of desaturation and chain-elongation reactions, this conversion is inefficient. Therefore, the consumption of fatty fish remains the principal dietary means by which humans obtain omega-3 PUFAs (9).

Omega-3 PUFAs have attracted considerable attention because of their extensive health benefits. In the cardiovascular system, omega-3 PUFAs have been reported to exert anti-inflammatory, vasodilatory, antiarrhythmic, antihypertensive, anticoagulant, and triglyceride-lowering effects, supporting their potential role in cardiovascular protection (10, 11). In the nervous system, omega-3 PUFAs are important components of neuronal cell membranes and may contribute to membrane fluidity, oxidative stress regulation, inflammatory modulation, and neuroprotection (12, 13). Adequate omega-3 PUFAs intake is also associated with perinatal health, including fetal brain and retinal development and a reduced risk of adverse pregnancy-related outcomes (14–17). In addition, their anti-inflammatory properties have been investigated in chronic inflammatory and metabolic diseases, such as asthma, psoriasis, inflammatory bowel disease, rheumatoid arthritis, and diabetes (18, 19). Emerging evidence also suggests that omega-3 PUFAs may be involved in biological aging, partly through their effects on inflammation and epigenetic regulation (20).

These broad medical effects provide an important biological basis for understanding the potential relevance of omega-3 PUFAs in oncology. Cancer development and progression are closely linked to chronic inflammation, oxidative stress, metabolic dysregulation, immune dysfunction, epigenetic alterations, and changes in cell signaling. Accordingly, omega-3 PUFAs have increasingly been investigated in cancer prevention, tumor progression, supportive care, and adjunctive therapy. Preclinical studies suggest that omega-3 PUFAs may inhibit tumor progression through multiple mechanisms, including the induction of apoptosis, regulation of lipid metabolism, epigenetic modulation, immune regulation, and remodeling of the tumor microenvironment (21–24). Clinical studies have further explored their potential value in improving quality of life and survival in patients with cancer cachexia, enhancing chemotherapy sensitivity, and reducing treatment-related adverse effects (25–29). However, despite extensive research, studies on omega-3 PUFAs in oncology remain heterogeneous and fragmented, spanning basic mechanistic investigations, epidemiological studies, biomarker-based assessments, nutritional interventions, perioperative immunonutrition, and combination strategies with anticancer therapies. Therefore, a systematic synthesis of the knowledge structure, research hotspots, and emerging trends in this field is needed to better characterize its current landscape and guide future translational research.

Bibliometrics provides a quantitative approach for mapping knowledge structures, research hotspots, and emerging frontiers through analyses of publication output, citation networks, keyword co-occurrence, and related indicators (30). In this study, bibliometric and visualization analyses were employed to systematically evaluate omega-3 PUFA-related cancer research from 2000 to 2025, with the aim of clarifying its research landscape, thematic evolution, and translational trends, thereby providing evidence-based insights for future basic research, nutritional oncology, and clinical practice.

## 2 Materials and methods

### 2.1 Literature sources and retrieval strategies

Data for this study were retrieved from the Web of Science Core Collection (WoSCC), Scopus, and PubMed databases on December 15, 2025. The following search strategies were used:

For the WoSCC database, the search formula was as follows:

((((((((TI = (cancer* OR tumor* OR oncology OR neoplasm* OR neoplasia* OR maliganc* OR carcinoma*)) OR AB = (cancer* OR tumor* OR oncology OR neoplasm* OR neoplasia* OR maliganc* OR carcinoma*)) OR AK = (cancer* OR tumor* OR oncology OR neoplasm* OR neoplasia* OR maliganc* OR carcinoma*)) AND TI = (“omega-3 PUFA*” OR “omega-3 fatty acid*” OR “omega-3 polyunsaturated fatty acid*” OR “omega-3 oil*” OR “n-3 PUFA*” OR “n-3 fatty acid*” OR “n-3 polyunsaturated fatty acid*” OR “ω-3 PUFA*” OR “ω-3 fatty acid*” OR “ω-3 polyunsaturated fatty acid*”)) OR AB = (“omega-3 PUFA*” OR “omega-3 fatty acid*” OR “omega-3 polyunsaturated fatty acid*” OR “omega-3 oil*” OR “n-3 PUFA*” OR “n-3 fatty acid*” OR “n-3 polyunsaturated fatty acid*” OR “ω-3 PUFA*” OR “ω-3 fatty acid*” OR “ω-3 polyunsaturated fatty acid*”)) OR AK = (“omega-3 PUFA*” OR “omega-3 fatty acid*” OR “omega-3 polyunsaturated fatty acid*” OR “omega-3 oil*” OR “n-3 PUFA*” OR “n-3 fatty acid*” OR “n-3 polyunsaturated fatty acid*” OR “ω-3 PUFA*” OR “ω-3 fatty acid*” OR “ω-3 polyunsaturated fatty acid*”)) AND DOP = (2000-01-01/2025-12-15)) AND DT = (Article OR Review)) AND LA = (English). For the Scopus database, the search formula was as follows:

(TITLE-ABS-KEY (cancer* OR tumor* OR oncology OR neoplasm* OR neoplasia* OR maliganc* OR carcinoma*)) AND (TITLE-ABS-KEY(“omega-3 PUFA*” OR “omega-3 fatty acid*” OR “mega-3 polyunsaturated fatty acid*” OR “omega-3 oil*” OR “n-3 PUFA*” OR “n-3 fatty acid*” OR “n-3 polyunsaturated fatty acid*” OR “ω-3 PUFA*” OR “ω-3 fatty acid*” OR “ω-3 polyunsaturated fatty acid*”)) AND PUBYEAR > 1999 AND PUBYEAR < 2026 AND (LIMIT-TO (DOCTYPE, “ar”) OR LIMIT-TO (DOCTYPE, “re”)) AND (LIMIT-TO (LANGUAGE, “English”)).

For the PubMed database, the search formula was as follows:

(cancer*[Title/Abstract] OR tumor*[Title/Abstract] OR oncology[Title/Abstract] OR neoplasm*[Title/Abstract] OR neoplasia*[Title/Abstract] OR maliganc*[Title/Abstract] OR carcinoma*[Title/Abstract]) AND (“omega-3 PUFA*”[Title/Abstract] OR “omega-3 fatty acid*”[Title/Abstract] OR “omega-3 polyunsaturated fatty acid*”[Title/Abstract] OR “omega-3 oil*”[Title/Abstract] OR “n-3 PUFA*”[Title/Abstract] OR “n-3 fatty acid*”[Title/Abstract] OR “n-3 polyunsaturated fatty acid*”[Title/Abstract] OR “ω-3 PUFA*”[Title/Abstract] OR “ω-3 fatty acid*”[Title/Abstract] OR “ω-3 polyunsaturated fatty acid*”[Title/Abstract]) Filters: Clinical Trial, English, from 2000 to 2025.

The literature retrieval and screening procedures were performed according to a predefined search strategy and eligibility criteria. Records identified from the Web of Science Core Collection were exported in plain-text format as full records, including cited references, whereas records retrieved from Scopus were exported in CSV format with complete bibliographic information, including cited references. Duplicate records were removed within each database separately based on DOI matching. Because clinical trial records are not comprehensively captured in WoSCC or Scopus, additional clinical trial information was retrieved from PubMed. Two independent researchers screened the retrieved clinical trial records by reviewing titles, abstracts, and keywords to exclude studies unrelated to omega-3 PUFAs and cancer. Discrepancies were resolved through discussion, and, when necessary, a third researcher was consulted for final adjudication. The complete search strategy is presented in Supplementary Figure S1.

### 2.2 Data analysis

Due to differences in data formats between the WoSCC and Scopus databases, data integration may lead to information loss. Therefore, separate analyses were conducted using datasets retrieved from each database to obtain more reliable findings. Given the standardized record format, relatively complete citation information, and broad compatibility of the WoSCC with commonly used bibliometric tools, the WoSCC dataset was used as the primary source for detailed bibliometric analyses, while Scopus data were used as a complementary dataset. Keyword clustering analysis and annual publication trends based on the Scopus database are presented in Supplementary material. Annual publication trends were evaluated using Origin 2018. Data were then subjected to visualization and scientific knowledge mapping using R software version 4.5.2, the bibliometrix package version 4.0 (31), VOSviewer version 1.6.20 (32), and CiteSpace version 6.4.R1 (33). To confirm data reliability and accuracy, data collection and analytical management were independently performed by two researchers.

VOSviewer was used to map co-authorship networks at both the national and institutional levels, perform source co-citation analysis, and examine keyword co-occurrence patterns. For network-based analysis of WoSCC author partnerships, the following thresholds were applied: (1) countries were included only if they had contributed at least five documents; (2) organizations were required to have published a minimum of 10 papers; (3) in the source co-citation analysis, sources were considered only if they had received no fewer than 300 citations; and (4) in the keyword co-occurrence analysis, the inclusion criterion was set at a minimum of 28 occurrences per keyword, with terms such as “omega-3 PUFAs” and “cancer” explicitly excluded. In the co-occurrence analysis of keywords from the Scopus database, keywords were required to occur at least 23 times, excluding search terms as well as their synonyms. The thresholds were determined through preliminary network testing to generate interpretable maps while retaining the major contributors and high-frequency terms. Generic search terms and their direct synonyms were excluded because their high frequency was driven by the search strategy rather than by thematic relevance. Journal impact factors used in this study were obtained from the 2024 edition of Journal Citation Reports (JCR).

## 3 Results

### 3.1 General landscape of global publications

**FIGURE 1:**
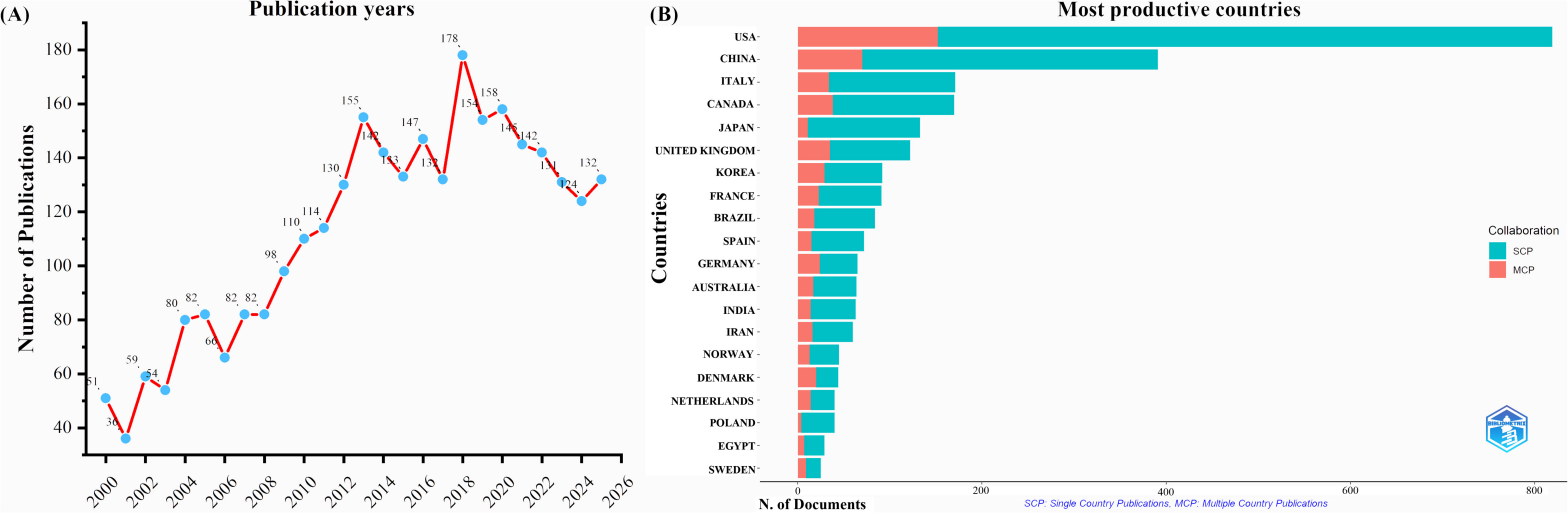
Trends in annual publication outputs on omega-3 PUFAs in cancer (2000–2025). **(A)** Yearly publication trends. **(B)** Distribution of corresponding authors’ countries and collaborations.

The WoSCC database yielded 2,917 publications after duplicates were removed. The annual publication volume showed an overall increasing trend from 2000 to 2025, with a notable peak around 2018 (Figure 1A). After duplicates were removed, the Scopus database yielded 7,380 distinct records. The publication growth trend in Scopus mirrored that observed in the WoSCC database (Supplementary Figure S2). This trend suggests that omega-3 PUFA-related cancer research has received sustained attention and has gradually developed into a stable research field.

**FIGURE 2:**
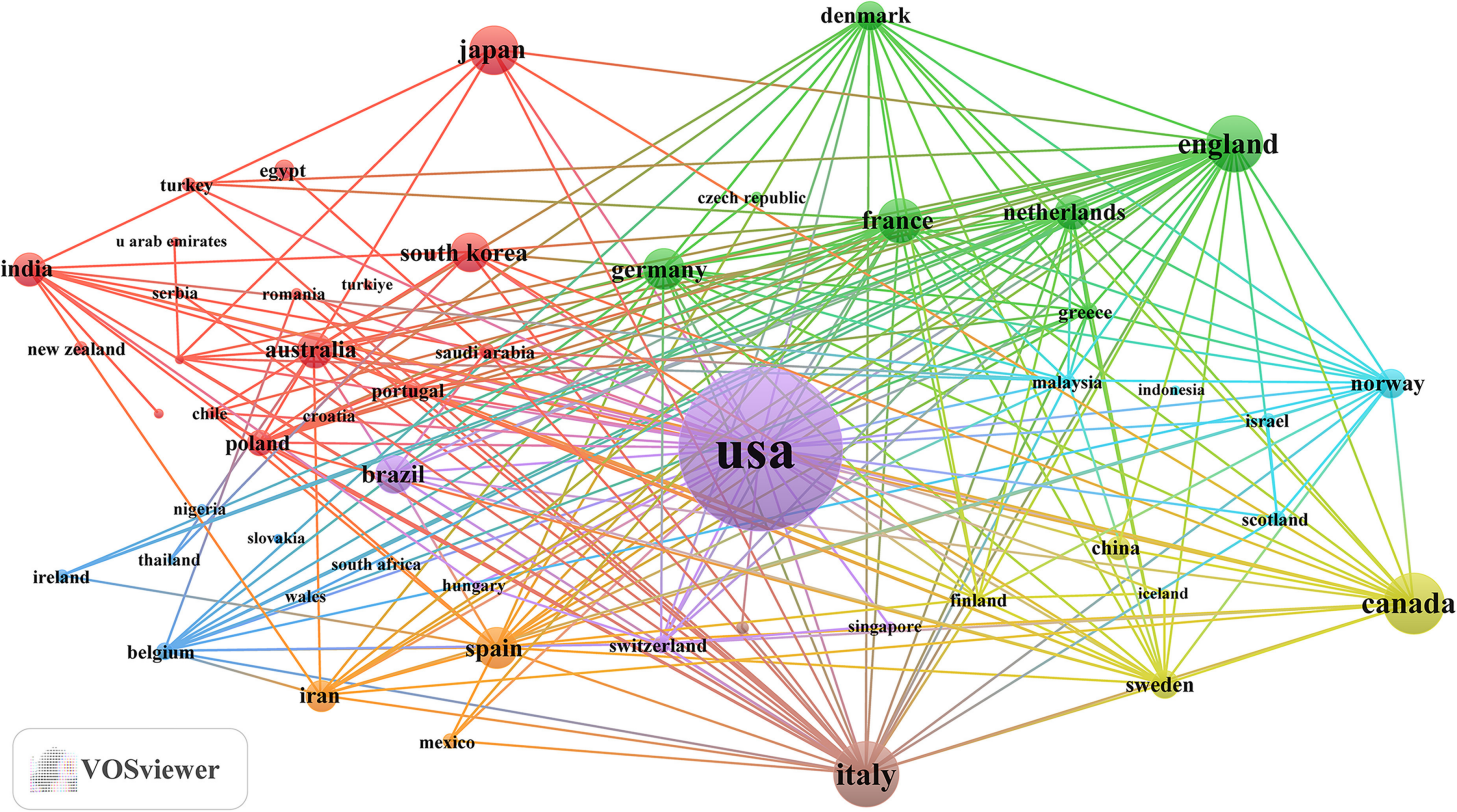
Map of cooperation between different countries involved in omega-3 PUFAs in cancer.

**FIGURE 3:**
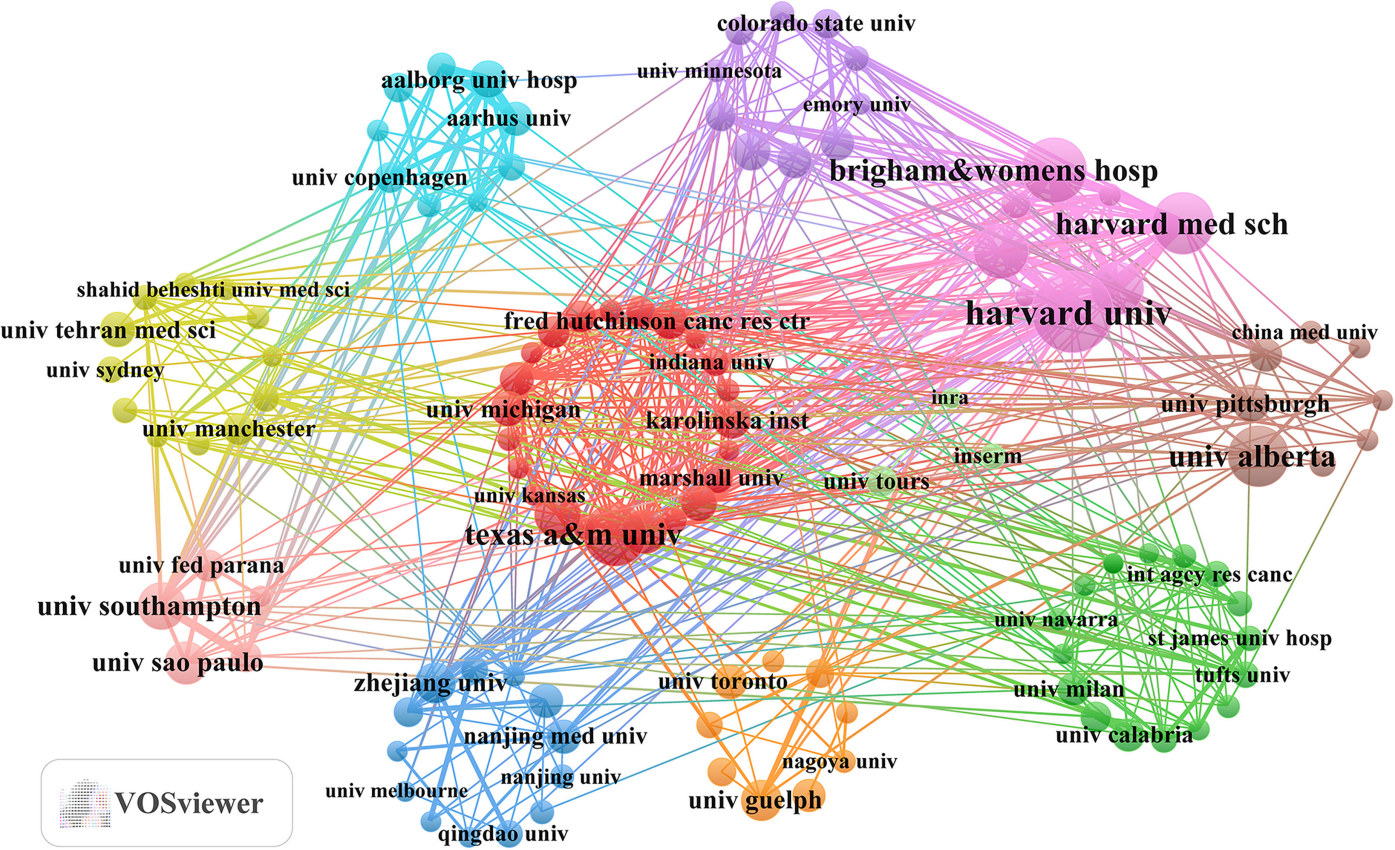
Map of collaboration among various institutions involved in omega-3 PUFAs in cancer.

Analysis of the corresponding authors’ countries revealed that the USA (*n* = 819) was the leading contributor, followed by China (*n* = 391) and Italy (*n* = 171), as shown in Figure 1B and Table 1. This pattern indicates that omega-3 PUFA-related cancer research has become a globally active field, with contributions from both Western and Asian research communities. Additionally, 18.6% of publications from the USA and 17.9% of publications from China involved multi-country collaborations (MCPs). In comparison, Denmark, Germany, and Korea had lower overall publication volumes; however, they achieved notable MCP rates of 45.5, 36.9, and 31.5%, respectively. As shown in Figure 2, the United States not only ranked first in terms of publication output but also maintains the most extensive network of international partnerships. Furthermore, Harvard University (*n* = 88) and Brigham and Women’s Hospital (*n* = 61) were identified as the core contributing institutions within the collaboration network (Figure 3 and Table 2). These findings suggest that academic influence in this field is shaped not only by publication output but also by collaborative connectivity.

**TABLE 1:** Corresponding authors in areas related to omega-3 PUFAs and cancer in the most relevant countries.

Country	Articles	SCP	MCP	Freq	MCP ratio
USA	819	667	152	28.1	18.6
China	391	321	70	13.4	17.9
Italy	171	137	34	5.9	19.9
Canada	170	132	38	5.8	22.4
Japan	133	122	11	4.6	8.3
United Kingdom	122	87	35	4.2	28.7
Korea	92	63	29	3.2	31.5
France	91	68	23	3.1	25.3
Brazil	84	66	18	2.9	21.4
Spain	72	57	15	2.5	20.8
Germany	65	41	24	2.2	36.9
Australia	64	47	17	2.2	26.6
India	63	49	14	2.2	22.2
Iran	60	44	16	2.1	26.7
Norway	45	32	13	1.5	28.9
Denmark	44	24	20	1.5	45.5
Netherlands	40	26	14	1.4	35
Poland	40	36	4	1.4	10
Egypt	29	22	7	1	24.1
Sweden	25	16	9	0.9	36
Portugal	20	16	4	0.7	20
Greece	17	13	4	0.6	23.5
Mexico	16	11	5	0.5	31.3
Switzerland	16	11	5	0.5	31.3
Turkey	16	12	4	0.5	25

MCP, multiple country publication; SCP, single country publication.

**TABLE 2:** Most relevant affiliations of omega-3 PUFAs and cancer.

Affiliation	Articles(n)
Harvard University	88
Brigham and Women’s Hospital	61
Texas A&M University	58
Harvard Medical School	57
University of Alberta	55
Massachusetts General Hospital	48
Harvard T.H. Chan School of Public Health	45
University of Southampton	38
University of Illinois	36
The Ohio State University	34
University of São Paulo	32
Zhejiang University	30
University of Guelph	29
University of California, Los Angeles	27
Aalborg University Hospital	26
Karolinska Institutet	26
University of Pittsburgh	26
Fred Hutchinson Cancer Research Center	25
National Cancer Institute	25
University of California, Davis	24

### 3.2 Journals and co-cited journals

To identify the most influential journals in omega-3 PUFA-related cancer research, we performed a bibliometric analysis using the Bibliometrix package in R software. Visualizations were generated using ggplot2 in R, and journal co-citation networks were analyzed and visualized using VOSviewer.

**FIGURE 4:**
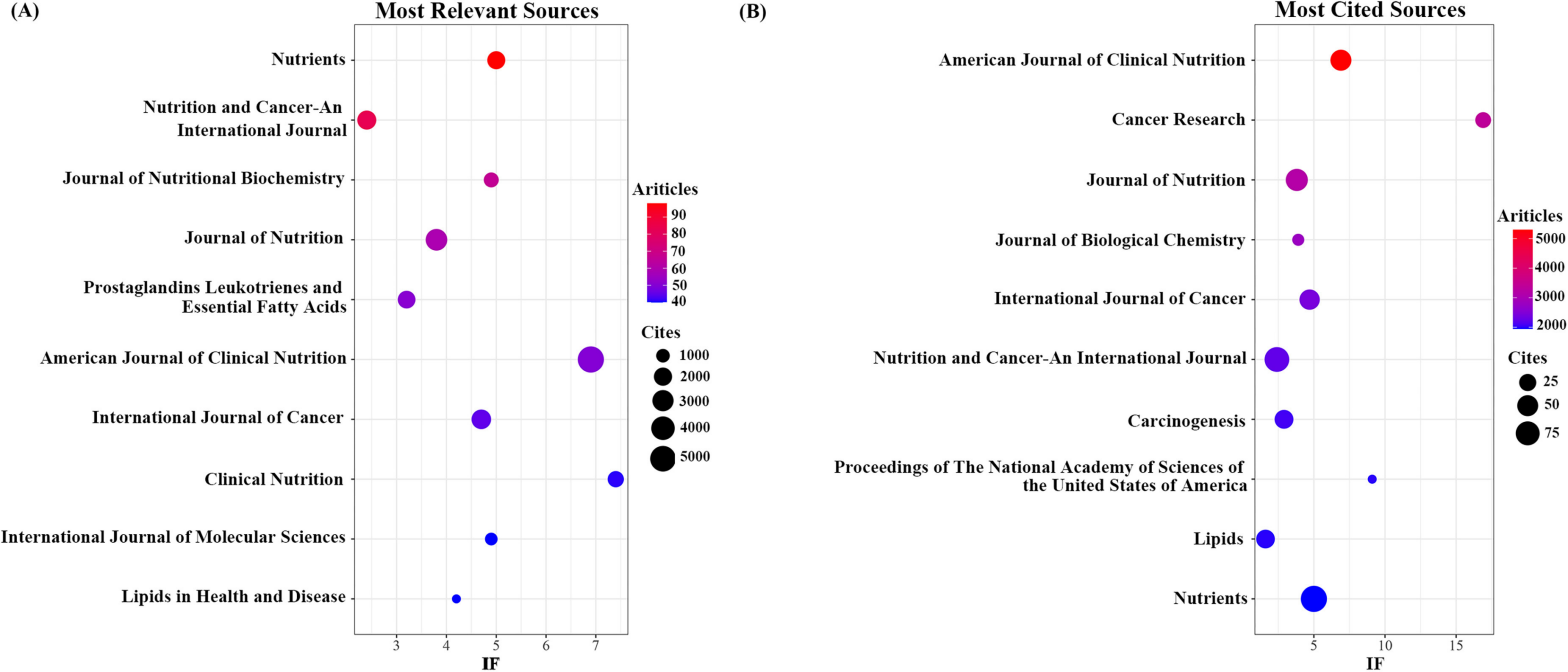
Journals with the most publications and the most citations. **(A)** Journals with the most publications. **(B)** Journals with the most citations.

This analysis identified 2,917 publications across 869 journals, with detailed data provided in Supplementary Annex 1. According to the data presented in Table 3 and visualized in Figure 4A, *Nutrients* (*n* = 98, IF = 5.0) was the most productive journal by publication volume, followed by *Nutrition and Cancer-An International Journal* (*n* = 83, IF = 2.4) and the *Journal of Nutritional Biochemistry* (*n* = 66, IF = 4.9). Meanwhile, the journals receiving the highest citation counts, as summarized in Table 4 and illustrated in Figure 4B, were led by the *American Journal of Clinical Nutrition* (*n* = 5,299, IF = 6.9), followed by *Cancer Research* (*n* = 3,376, IF = 16.9) and the *Journal of Nutrition* (*n* = 3,177, IF = 3.8).

**TABLE 3:** Top 10 productive journals related to omega-3 PUFAs in cancer.

Journal	Documents	IF(2024)	Cites
Nutrients	98	5	1947
Nutrition and Cancer-An International Journal	83	2.4	2246
Journal of Nutritional Biochemistry	66	4.9	1223
Journal of Nutrition	59	3.8	3177
Prostaglandins Leukotrienes and Essential Fatty Acids	51	3.2	1860
American Journal of Clinical Nutrition	50	6.9	5299
International Journal of Cancer	45	4.7	2429
Clinical Nutrition	41	7.4	1486
International Journal of Molecular Sciences	40	4.9	879
Lipids in Health and Disease	40	4.2	664

**TABLE 4:** Top 10 most cited journals related to omega-3 PUFAs in cancer.

Journal	Cites	IF(2024)	Documents
American Journal of Clinical Nutrition	5299	6.9	50
Cancer Research	3376	16.9	19
Journal of Nutrition	3177	3.8	59
Journal of Biological Chemistry	2813	3.9	7
International Journal of Cancer	2429	4.7	45
Nutrition and Cancer-An International Journal	2246	2.4	83
Carcinogenesis	2081	2.9	35
Proceedings of The National Academy of Sciences of the United States of America	2005	9.1	4
Lipids	1998	1.6	34
Nutrients	1947	5	98

**FIGURE 5:**
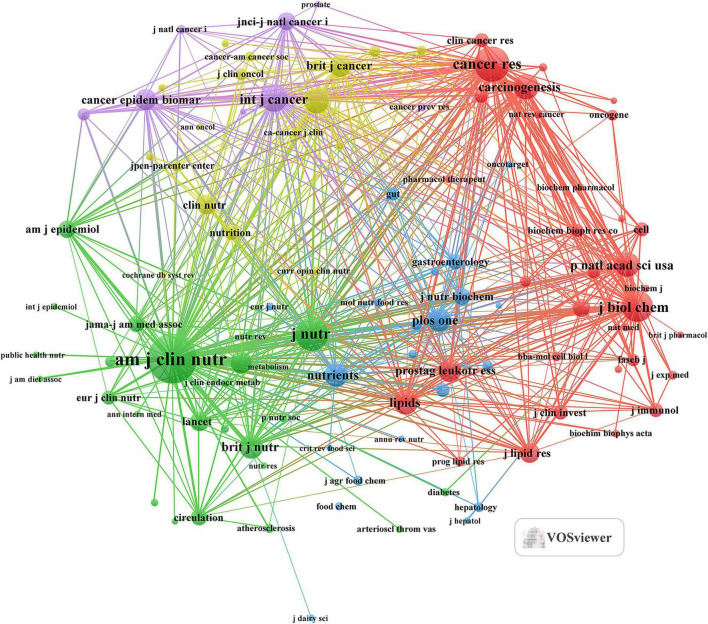
Co-citation journals related to omega-3 PUFAs in cancer.

The journal co-citation analysis (Figure 5) identified the American Journal of Clinical Nutrition, Cancer Research, International Journal of Cancer, and Nutrients as core journals forming a collaborative nexus that anchors intellectual exchange within this research domain. Collectively, these results underscore the pivotal role of the American Journal of Clinical Nutrition in omega-3 PUFA-related cancer research. Furthermore, the data indicate a notable publication gap in high-impact journals within this domain. This disparity highlights a critical opportunity to enhance the academic impact and methodological rigor of future studies.

### 3.3 Citation burst

**FIGURE 6:**
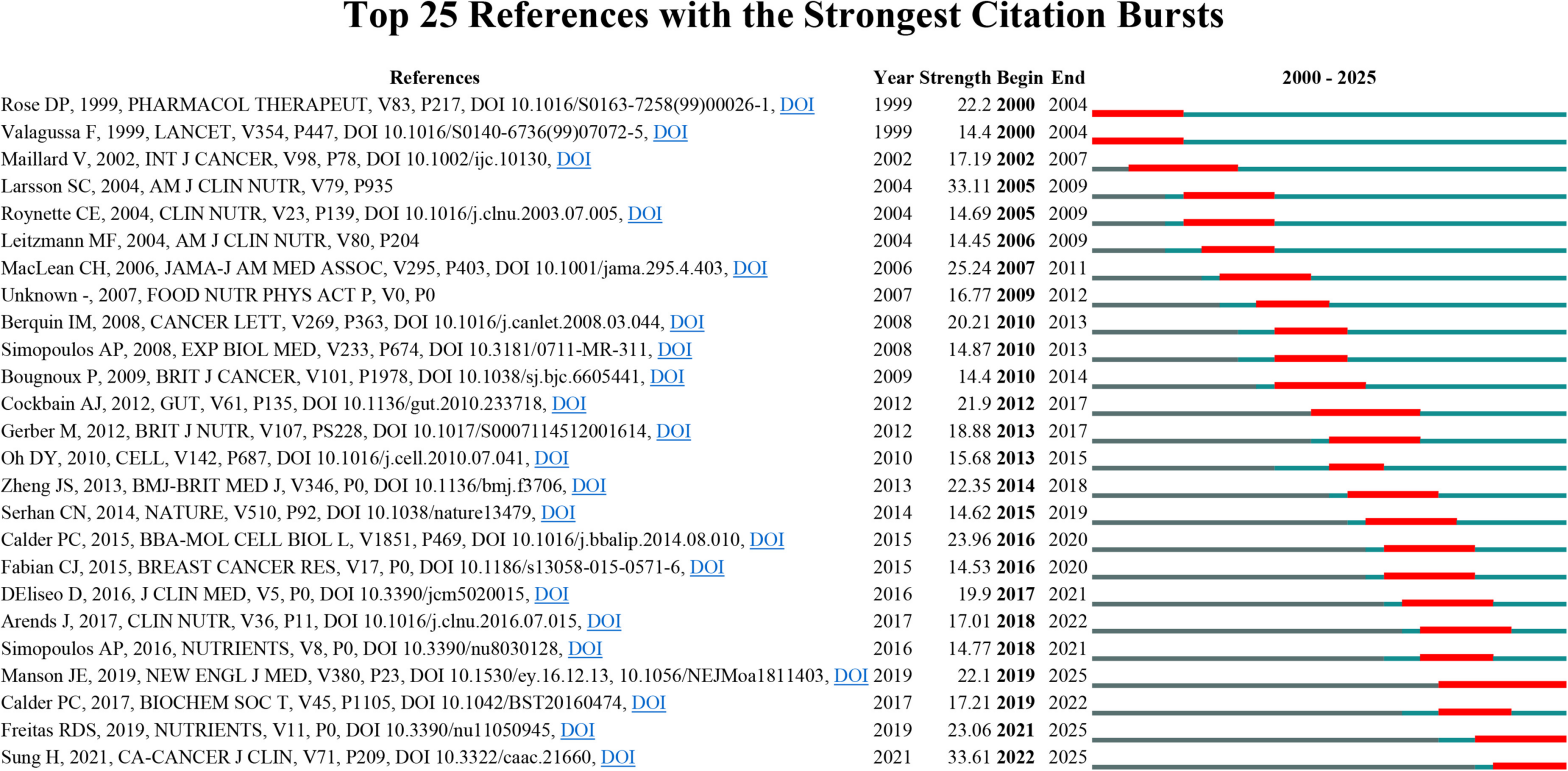
Top 25 references with the strongest citation bursts in omega-3 PUFAs.

To explore the frontiers and key research domains related to omega-3 PUFAs in cancer, CiteSpace was employed to identify the 25 most prominent citation bursts in omega-3 PUFA-related cancer research (Figure 6). The corresponding article titles and Digital Object Identifiers (DOIs) are provided in Supplementary Annex 2. Notably, the three publications with the strongest citation burst intensities were as follows: (1) “Global Cancer Statistics 2020: GLOBOCAN Estimates of Incidence and Mortality Worldwide for 36 Cancers in 185 Countries” (strength: 33.61); (2) “Dietary Long-Chain Omega-3 PUFAs for the Prevention of Cancer: A Review of Potential Mechanisms” (strength: 33.11); and (3) “Effects of Omega-3 Fatty Acids on Cancer Risk: A Systematic Review” (strength: 25.24). Furthermore, the titles reflecting the most recent and emerging areas of strong citation activity include: (1) “Omega-3 Fatty Acids and Inflammatory Processes: From Molecules to Man”; (2) “Protective Effects of Omega-3 Fatty Acids in Cancer-Related Complications”; and (3) “Global Cancer Statistics 2020: GLOBOCAN Estimates of Incidence and Mortality Worldwide for 36 Cancers in 185 Countries.”

Overall, citation burst analysis identified three core research directions for omega-3 PUFAs in oncology: (1) multi-target anticancer molecular mechanisms, focusing on elucidating the biological basis underlying their antitumor effects through the regulation of lipid metabolism, inflammatory signaling pathways, and apoptosis; (2) the epidemiological significance of omega-3 PUFAs in cancer and other chronic diseases, exploring the role of fatty acid balance as a key dietary factor in disease risk assessment and prevention; and (3) the translational application of omega-3 PUFAs in clinical cancer management, systematically analyzing their potential value and practical progress as immunonutrients in adjuvant therapy, complication control, and improvement of patient outcomes.

### 3.4 Keyword clusters and evolution

Keyword clustering analysis facilitates a rapid understanding of the research landscape and core thematic directions within a specific field. In this study, 9,908 keywords were extracted from the literature using VOSviewer. The top 20 keywords with occurrence frequencies exceeding 130, which collectively reflect the central research hotspots within the domain, are listed in Table 5. Among these, the keyword “inflammation” ranked highest with a frequency of 465, followed by “breast cancer” (*n* = 382), “risk” (*n* = 336), “colorectal cancer” (*n* = 286), “expression” (*n* = 283), and “apoptosis” (*n* = 268).

**TABLE 5:** The top 20 keywords in omega-3 PUFAs and cancer.

Rank	Words	Counts
1	Inflammation	465
2	Breast cancer	382
3	Risk	336
4	Colorectal cancer	286
5	Expression	283
6	Apoptosis	268
7	Growth	221
8	Colon cancer	206
9	Supplementation	206
10	Oxidative stress	205
11	Cardiovascular disease	195
12	Alpha-linolenic acid	191
13	Diet	183
14	Double-blind	166
15	Prevention	161
16	Cells	157
17	Nutrition	150
18	Fish	145
19	Prostate cancer	137
20	Arachidonic acid	134

**FIGURE 7:**
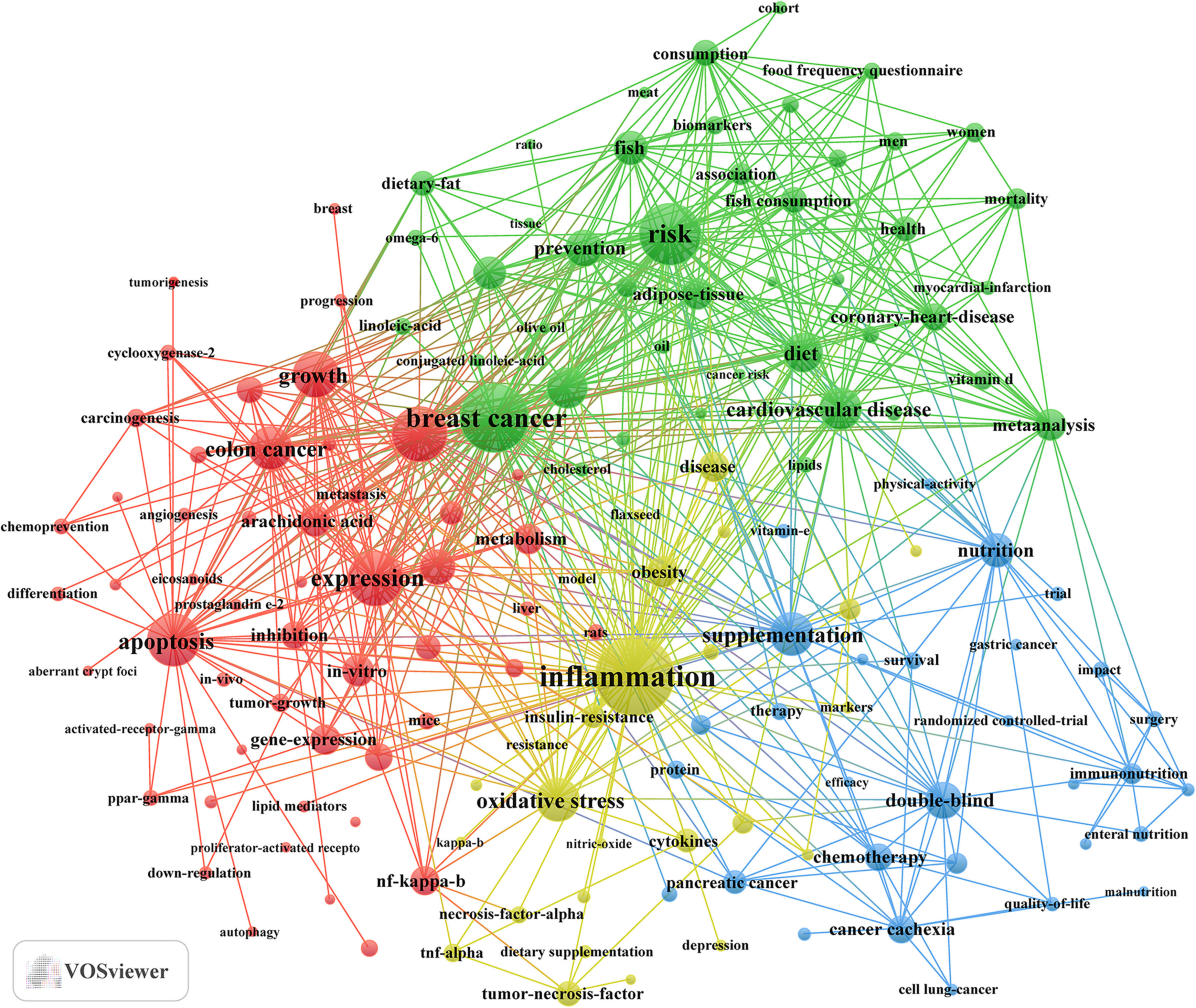
Visualization of keywords on research of omega-3 PUFAs in cancer.

Through cluster analysis, four distinct colored clusters were identified, as shown **in** Figure 7: (1) The multi-target molecular mechanisms and preclinical evidence of omega-3 PUFAs in colorectal cancer chemoprevention and treatment (red): This cluster comprises 52 keywords, including colorectal cancer, apoptosis, NF-kappa-B, and PPAR-gamma, among others. (2) Population-level evidence on the correlation between dietary omega-3 PUFAs exposure and chronic disease incidence, with a focus on breast cancer, prostate cancer, and cardiovascular diseases (green): This cluster contains 44 keywords, such as breast cancer, prostate cancer, risk, diet, and meta-analysis. (3) Clinical efficacy evaluation of omega-3 PUFAs nutritional interventions in cancer treatment (blue): This cluster includes 30 keywords, such as malnutrition, randomized controlled trial, supplementation, cancer cachexia, and enteral nutrition. (4) The role of omega-3 PUFAs in modulating metabolic diseases and the tumor microenvironment via inflammation and oxidative stress pathways (yellow): This cluster contains 26 keywords, including inflammation, oxidative stress, cytokines, insulin resistance, lipid metabolism, and gut microbiota. All keywords across the four clusters are detailed in Supplementary Annex 3.

In addition, VOSviewer was used to extract and analyze keywords from Scopus (Supplementary Figure S3). Based on an in-depth analysis of the Scopus database, we have summarized the following four research hotspots: (1) Omega-3 PUFAs directly induce tumor cell apoptosis and suppress malignant behaviors such as proliferation and metastasis by modulating specific lipid mediators and inflammatory pathways, including NF-κB signaling; (2) By ameliorating metabolic disorders related to “obesity-insulin resistance,” omega-3 PUFAs serve as a preventive or adjunctive therapeutic strategy for associated cancers; (3) As a core component of immunonutrition, omega-3 PUFAs are clinically utilized to manage cancer cachexia, optimize body composition, and enhance the synergistic efficacy of radiotherapy, chemotherapy, and surgical interventions; (4) Epidemiological studies have demonstrated that omega-3 PUFAs synergistically reduce the incidence and risk of various malignancies, including colorectal cancer, as well as cardiovascular diseases.

**FIGURE 8:**
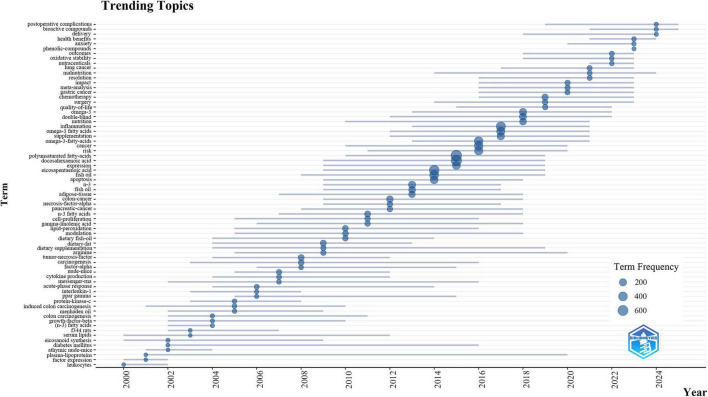
Trending topics of omega-3 PUFAs in cancer.

This study employed the Bibliometrix toolkit to conduct a temporal dynamic analysis of the thematic evolution of omega-3 PUFAs in cancer research (Figure 8). The annual frequencies of all terms and their evolutionary relationships are provided in Supplementary Annex 4. The research trends demonstrate a clear chronological progression: From 2005 to 2011, studies primarily focused on fundamental biochemical mechanisms and fatty acid metabolic pathways. Core keywords included omega-3 fatty acids, polyunsaturated fatty acids, and lipid peroxidation, with an emphasis on exploring anti-inflammatory and antioxidant mechanisms in cellular and animal models. During 2012–2017, research gradually shifted toward clinical translation and the management of cancer-related symptoms. Keywords such as dietary supplementation, chemotherapy-related fatigue, quality of life, and postoperative complications emerged as prominent themes, reflecting a transition from basic mechanistic research to clinical nutritional interventions. Between 2018 and 2021, research further expanded into metabolic regulation and its interaction with the tumor immune microenvironment. Keywords such as inflammation, oxidative stress, gut microbiota, and metabolic syndrome showed significant increases, indicating that systemic metabolic regulation and its interplay with the tumor immune microenvironment are emerging research foci. Since 2022, the field has shifted toward precision intervention and multidisciplinary integration. Key areas of focus include personalized nutritional support strategies, mechanisms of tumor metabolic reprogramming, the integrated application of multi-omics technologies, and the synergistic effects of omega-3 PUFAs with immunotherapy and targeted therapies. This reflects a deepening integration between clinical practice and mechanistic research. This thematic evolution suggests that future research will increasingly emphasize molecular mechanism-driven precision nutrition strategies, exploration of mechanisms underlying multimodal therapeutic synergies, and the advancement of clinical translation through real-world evidence.

### 3.5 Comprehensive analysis of hotspots

In summary, through citation burst analysis, keyword clustering, keyword frequency analysis, and thematic evolution analysis of the WoSCC database, as well as keyword clustering analysis of the Scopus database, we identified key research hotspots at the intersection of omega-3 PUFAs and cancer research. These findings reveal that published studies have primarily focused on three thematic clusters: (1) investigation of the multi-target molecular mechanisms underlying the antitumor effects of omega-3 PUFAs; (2) assessment of the link among dietary intake of omega-3 PUFAs, corresponding biomarker levels, and cancer risk; and (3) exploration and efficacy evaluation of omega-3 PUFAs in clinical oncology treatment.

### 3.6 Clinical progress analysis

Based on the PubMed database, 167 clinical trials were systematically screened and identified. Three major research themes were broadly categorized in this study: (1) the role of omega-3 PUFAs as metabolic and immune microenvironment modulators in chemoprevention among high-risk populations; (2) the integration of omega-3 PUFAs as systemic regulators of energy metabolism and inflammation in supportive care throughout the cancer continuum; (3) the role of omega-3 PUFAs in enhancing therapeutic efficacy through multimodal combination therapy.

## 4 Discussion

### 4.1 General information

We retrieved 2,917 relevant publications focusing on omega-3 PUFAs and tumor research from the WoSCC database. The annual publication volume in this field showed an overall upward trend from 2000 to 2018, followed by a decline and gradual stabilization. The publication peak around 2018 likely reflects the concentrated emergence of mechanistic and early translational research findings over the past decade (34–36), coinciding with heightened academic interest in the roles of chronic inflammation and lipid metabolism in cancer progression (37). This growth aligns with the broader context of increased research funding in nutritional oncology and heightened attention to interdisciplinary studies during that period. However, the subsequent plateau and slight decline in publication volume after 2018 may be partly explained by several broader contextual factors, including mixed findings from large randomized trials, increasing attention to emerging oncology fields such as immunotherapy and microbiome research, and disruptions to non-COVID-related clinical research during the COVID-19 pandemic; however, these interpretations should be considered exploratory rather than causal (38–43). Despite the recent slowdown in publication growth, the sustained attention to this field over more than two decades underscores the enduring significance of omega-3 PUFAs in cancer research (44).

In the field of omega-3 PUFAs and cancer research, the United States leads in publication output while maintaining the most extensive international collaboration network. China ranks second in publications and also demonstrates strong collaborative engagement. Interestingly, countries with relatively lower overall output, such as Denmark, Germany, and South Korea, exhibit high levels of international collaboration. This suggests that smaller research communities may enhance their influence and access resources through cooperation. Among the top 20 research institutions by publication volume, Harvard University, Brigham and Women’s Hospital, and Texas A&M University in the United States stand out, underscoring the dominance of the United States in this field. Concurrently, institutions from China, Canada, the United Kingdom, Brazil, Denmark, and Sweden also hold significant positions, reflecting the global attention this research area has garnered and the increasingly close international collaborative efforts.

The 2,917 included studies were published in 869 journals, with *Nutrients, Nutrition and Cancer*, *Journal of Nutritional Biochemistry* contributing significantly through high publication volumes. Additionally, the *American Journal of Clinical Nutrition* and *Cancer Research* rank as the two most frequently cited journals in this field. Notably, based on a comprehensive evaluation of indicators, including impact factor, citation frequency, and publication volume, the *American Journal of Clinical Nutrition* has been identified as the core journal in this research domain.

### 4.2 Hot topics and development trends

A multidimensional bibliometric analysis—integrating citation impact, keyword co-occurrence, thematic evolution, and clinical trial landscapes—converges to define three principal research fronts in omega-3 PUFAs and oncology.

#### 4.2.1 Research on the multi-target molecular mechanisms of omega-3 PUFAs’ anti-tumor effects

Keyword cluster analysis indicates that “inflammation,” “apoptosis,” “NF-κB,” “PPAR-γ,” “oxidative stress,” and “insulin resistance” have been identified as key mediators of the antitumor effects of omega-3 PUFAs. By competitively engaging metabolic pathways and directly regulating cellular signaling networks, they demonstrate significant anti-tumor potential. Omega-3 PUFAs can remodel the tumor immune microenvironment, shifting it from a pro-inflammatory state to an anti-inflammatory or inflammation-resolving state. Specifically, Omega-3 PUFAs inhibit lipopolysaccharide-induced production of pro-inflammatory cytokines (TNF-α, IL-6, and IL-1β) through modulation of the NF-κB signaling pathway (45, 46). Clinically, they also reduce circulating levels of IL-6 and TNF in cancer patients (46). Omega-3 PUFAs also compete with n-6 PUFAs for metabolic pathways, leading to decreased production of pro-inflammatory eicosanoids (e.g., prostaglandins and leukotrienes) while generating anti-inflammatory lipid mediators such as resolvins and protectins, ultimately promoting the resolution of inflammation (47). Additionally, omega-3 PUFAs are incorporated into cell membrane phospholipids, altering the physicochemical properties of membranes. This modification reshapes the structure and function of lipid rafts, thereby regulating signaling cascades involved in proliferation, apoptosis, and metastasis (48). Omega-3 PUFAs also promote tumor cell apoptosis through multiple pathways. For example, in acute myeloid leukemia cells, they enhance oxidative stress by activating the Nrf2 pathway (49), while their derivative, 4-hydroxyhexenal, induces heme oxygenase-1, thereby exacerbating apoptosis (50). Furthermore, omega-3 PUFAs downregulate anti-apoptotic proteins, including Bcl-2 and procaspase-8, while concurrently upregulating Bax (51). Moreover, omega-3 PUFAs block the synthesis of pro-angiogenic arachidonate-like acids by reducing the release of membrane-bound arachidonic acid and competitively inhibiting cyclooxygenase (COX) and lipoxygenase (LOX) activity (47). They also hinder mast cell-mediated angiogenesis by disrupting the association of FcεRI with lipid rafts (52, 53), thus blocking pro-angiogenic signals through multiple mechanisms. Recent studies have further demonstrated that in acidic tumor microenvironments, omega-3 PUFAs selectively induce ferroptosis in cancer cells, thereby suppressing tumor growth (54). Additionally, omega-3 PUFAs have been recognized as chemotherapeutic sensitizers, enhancing the efficacy of carboplatin in ovarian cancer treatment (55), increasing osteosarcoma sensitivity to cisplatin (56), and promoting oxaliplatin-induced tumor cell apoptosis by upregulating SESN2 and CHOP expression (57).

Epigenetic regulation may provide an additional mechanistic explanation for the antitumor effects of omega-3 PUFAs. Tumor initiation and progression are shaped not only by genetic alterations but also by epigenetic dysregulation, including aberrant DNA methylation, histone modifications, chromatin remodeling, and non-coding RNA-mediated regulation of gene expression (58, 59). Existing evidence suggests that DHA may regulate apoptosis-related gene expression through promoter methylation-related mechanisms, particularly when combined with butyrate in colorectal cancer models (60). Omega-3 PUFAs may also upregulate TET1 expression, promote the conversion of 5-methylcytosine to 5-hydroxymethylcytosine, and increase genomic DNA hydroxymethylation, thereby contributing to colorectal cancer suppression (61). Importantly, histone modifications, particularly histone acetylation, also appear to be involved in PUFA-mediated epigenetic regulation. In a C3(1)-Tag mouse model, maternal omega-3-rich dietary exposure has been reported to reduce mammary tumorigenesis and, in related mechanistic analyses, to induce genome-wide histone modification changes in offspring mammary tissue, including increased H3K18 acetylation and altered H3K4me2 signals around transcription start sites (62, 63). These histone mark changes were associated with differential expression of genes enriched in pathways related to p53 signaling, DNA repair, inflammatory responses, apoptosis, and cell-cycle regulation (63). DHA has also been reported to alter histone acetylation patterns and increase p53 acetylation in diffuse large B-cell lymphoma cells, suggesting that omega-3 PUFAs may affect both chromatin-associated histone marks and acetylation of non-histone tumor suppressor proteins (64). In addition, omega-3 fatty acid supplementation has been shown to inhibit NF-κB activation, increase *ex vivo* sensitivity to doxorubicin, and downregulate genes associated with cancer promotion and malignant progression in early-stage chronic lymphocytic leukemia (65). Although this study did not directly examine chromatin marks, it supports the possibility that omega-3 PUFAs may reshape tumor-related inflammatory and transcriptional programs. Non-coding RNA-related regulation may represent another layer of this network, as DHA has been reported to modulate miR-126 promoter methylation and miR-126 expression, thereby affecting VEGF-mediated angiogenic signaling (66). Taken together, these findings suggest that omega-3 PUFAs may influence cancer biology through multilayered epigenetic mechanisms involving DNA methylation/demethylation, histone and non-histone protein modifications, and non-coding RNA-mediated regulation.

Despite extensive investigation into the antitumor effects of omega-3 PUFAs, further work is needed to elucidate their specific molecular targets across different tumor subtypes and facilitate their application in precision medicine (67). Additionally, further investigation of their synergistic mechanisms with chemotherapy or immunotherapy is required to provide a theoretical foundation for optimizing combination treatment strategies (68).

#### 4.2.2 Population-based evidence systems research on the association between dietary exposure to omega-3 PUFAs and cancer risk

Previous studies have examined the relationship between dietary omega-3 PUFAs intake (or its biomarker levels) and the incidence of various cancers is a current research hotspot. Evidence is particularly concentrated on colorectal, breast, and prostate cancers, though significant heterogeneity exists among population study results.

In colorectal cancer, multiple extensive cohort studies and meta-analyses indicate that while total dietary omega-3 PUFAs intake shows a weak association with risk, EPA and DHA demonstrate significant protective effects (69). Colorectal cancer risk decreased by 11 and 12%, respectively, in the groups with the highest intake of EPA and DHA; for each 0.1-g/day increase in EPA or DHA intake, the risk decreased by 5 and 3%, respectively (69). Blood biomarker studies reveal stronger associations: a 1% increase in serum omega-3 PUFA levels was associated with a 4% reduction in incidence risk, demonstrating a linear dose-response relationship (69). However, the results of existing studies exhibit heterogeneity. The EPIC cohort supports the association of higher total long-chain omega-3 PUFA intake and a lower n-6/n-3 ratio with reduced colorectal cancer risk (70). In contrast, the Japan Collaborative Cohort found no association with marine-derived omega-3 PUFAs, with only ALA showing a negative correlation with distal colon cancer risk (71). UK Biobank data suggested a nonlinear inverse association of plasma total omega-3 PUFAs and DHA with colorectal cancer risk, which was more pronounced in proximal colon cancer and among men (72). Collectively, these findings support a potential protective role of omega-3 PUFAs (particularly EPA and DHA) against colorectal cancer. For breast cancer, the evidence is more complex. Overall, dietary omega-3 PUFAs or ALA show mostly non-significant associations with breast cancer risk (73, 74), but some studies suggest that marine-derived long-chain omega-3 PUFAs (EPA/DHA) may offer protective effects (74). More objective biomarker studies indicate that circulating levels of long-chain omega-3 PUFAs are negatively correlated in relation to breast cancer risk, with this association being more pronounced in premenopausal women, hormone receptor-positive subtypes, and overweight/obese populations (75, 76). In prostate cancer research, conclusions remain inconclusive. Some studies suggest that a low dietary n-6/n-3 ratio may be linked to a decreased risk (77), but other large cohorts have not identified a clear association (78). Biomarker studies further reveal tissue specificity: EPA levels in prostate tissue were inversely associated with high-grade prostate cancer risk, whereas levels in circulating red blood cells show no such association (79). Furthermore, evidence linking omega-3 PUFAs to other cancers remains limited. The EPIC research revealed a marked inverse correlation between plasma phospholipid omega-3 PUFAs (particularly ALA and docosahexaenoic acid) and pancreatic cancer risk (80). Japanese cohort studies indicate that marine-derived omega-3 PUFAs may slightly reduce gastric cancer risk in Japanese women (81).

Overall, the association between omega-3 PUFAs and cancer incidence appears to be influenced by multiple factors, including the dietary omega-6/omega-3 PUFA ratio, which may competitively modulate the biological effects of omega-3 PUFAs (82), as well as the baseline nutritional status and genetic background of the study population (83), and the molecular and anatomical heterogeneity of tumors (72). In addition, dose–response relationships may partly explain the inconsistent findings across studies. Variations in dietary intake, supplement dosage, EPA/DHA composition, and circulating or tissue biomarker levels may influence the dose–response relationship and result in linear, nonlinear, or threshold associations between omega-3 PUFAs and cancer risk. Therefore, large-scale, well-designed prospective studies are needed to further clarify the relationship between omega-3 PUFAs and cancer development. Personalized intervention strategies should also be explored according to individual characteristics, such as baseline omega-3 PUFA status, obesity status, genetic background, and tumor molecular subtype. Furthermore, standardized biomarker assessment combined with dose–response modeling is required to clarify the specific effects of different omega-3 PUFAs, such as EPA and DHA, and their dynamic changes across different stages of tumorigenesis.

#### 4.2.3 Exploration of omega-3 PUFAs in clinical cancer treatment and efficacy evaluation

The exploration of omega-3 PUFAs in clinical cancer management has become a key focal point of scholarly inquiry in this field. Current research primarily focuses on three key areas: omega-3 PUFAs in nutritional support for cancer cachexia, optimization of perioperative immunonutrition, and synergistic effects and toxicity management in adjuvant therapy.

##### 4.2.3.1 Clinical application of omega-3 PUFAs in cancer cachexia

Cancer cachexia is a complex metabolic disorder characterized by progressive body mass loss, skeletal muscle atrophy and systemic inflammation, which significantly impairs patients’ quality of life and clinical prognosis. Omega-3 PUFAs have attracted considerable scholarly interest because of their potential role in the treatment of cancer cachexia owing to their anti-inflammatory and metabolic regulatory properties (84). Clinical evidence suggests that omega-3 PUFA supplementation may improve the overall wellbeing and functional status of patients. A meta-analysis involving 1,184 patients demonstrated that omega-3 PUFAs significantly improved wellbeing and quality of life and extended median survival. However, they exerted no significant effect on body mass or pro-inflammatory factor levels (25). Another meta-analysis focused on advanced non-small cell lung cancer with cachexia similarly reported omega-3 PUFA-induced improvements in quality of life and observed significant weight gain. However, no apparent effect on lean body mass or skeletal muscle mass was noted (85). A review synthesizing 57 independent oncology clinical trials further supported the prospective advantages of omega-3 PUFAs in maintaining body weight, improving survival, and modulating immune parameters (86). Although consistent conclusions concerning the impacts of omega-3 PUFAs on body composition (e.g., lean body mass) and specific inflammatory markers remain elusive, existing evidence supports omega-3 PUFAs supplementation as a safe and potentially beneficial adjunctive therapy for chemotherapy patients at risk of malnutrition and cachexia. This approach may help mitigate chemotherapy-related adverse reactions, enhance quality of life, and improve survival outcomes (87).

##### 4.2.3.2 Clinical application of omega-3 PUFAs in the perioperative period of cancer

Surgery remains the primary treatment for solid tumors. However, surgical trauma induces intense stress and systemic inflammatory responses, increasing the risk of postoperative infections and delaying recovery—an issue particularly pronounced in malnourished cancer patients. Multiple high-quality studies confirm that perioperative supplementation with omega-3 PUFAs significantly addresses this clinical challenge. A prospective, randomized controlled study conducted on patients with gastric cancer demonstrated that postoperative enteral and parenteral nutrition supplemented with omega-3 PUFAs significantly increased lymphocyte counts, CD3^+^ and CD4^+^ T cell levels, and the CD4^+^/CD8^+^ ratio (88). It also improved nutritional markers such as total protein and albumin, while reducing levels of CR, IL-6, and TNF-α, and decreasing the occurrence rate of postoperative complications (88). A large meta-analysis further consolidated evidence showing that omega-3 PUFAs significantly improve nutritional and immune function in gastrointestinal cancer patients, reduce systemic inflammation, lower the risk of leakage at the anastomosis site and incisional infections, and suggest an optimal supplementation dose of 0.16–0.30 g/kg/day (89). Additionally, in prostate cancer patients, supplementation with long-chain omega-3 PUFAs may improve urinary symptoms in those undergoing radical prostatectomy (90). However, one study showed that administering an oral nutrient preparation fortified with 2.0 g EPA and 1.0 g DHA daily from several days preoperatively to 7 days postoperatively in colorectal cancer patients did not significantly alter the incidence of postoperative complications (91). Although the substantial advantages of perioperative omega-3 PUFAs supplementation for cancer patients remain controversial, most studies suggest that they exhibit the propensity to improve immunity, reduce inflammation, and optimize specific clinical therapeutic results during the perioperative period. Therefore, integrating omega-3 PUFAs into the immunonutrition protocols of Enhanced Recovery After Surgery (ERAS) pathways holds crucial clinical value for optimizing the short- and long-term prognosis of surgical patients.

##### 4.2.3.3 Synergistic effects of omega-3 PUFAs with anticancer drugs and reduction of treatment-related toxicity

Chemotherapy, radiotherapy, and immunotherapy are core strategies in comprehensive cancer treatment, yet their efficacy is often limited by tumor resistance and treatment-related toxicity. Preclinical studies indicate that omega-3 PUFAs can enhance chemosensitivity through multiple mechanisms (45, 48, 51, 54), and emerging clinical evidence also supports their potential as adjunctive agents. In a randomized controlled study of locally advanced breast cancer, omega-3 PUFA supplementation combined with CAF neoadjuvant chemotherapy significantly reduced the expression intensity of the tumor cell proliferation marker Ki-67 and vascular endothelial growth factor (VEGF), while prolonging overall survival and disease-free survival (26). Another recent study indicated that, among patients with non-small cell lung cancer receiving first-line pembrolizumab, those with higher pretreatment serum arachidonic acid/eicosapentaenoic acid ratios showed better survival outcomes. This suggests that omega-3 PUFA supplementation may serve as a novel nutritional strategy to enhance the efficacy of immunotherapy (92). Recent reviews further indicate that combining omega-3 PUFAs with immunotherapy has emerged as a novel research direction in this field (68).

Regarding the management of treatment-related toxicities, omega-3 PUFAs demonstrate specific protective effects. Chemotherapy-induced peripheral neuropathy is a common toxicity that impacts quality of life, a comprehensive synthesis of existing research indicates that oral omega-3 PUFAs may reduce its risk (27). Owing to their anti-inflammatory properties, clinical studies have confirmed that omega-3 PUFAs can effectively prevent and manage chemotherapy-induced oral mucositis while alleviating associated pain (28). In patients with gastrointestinal cancer, fish oil supplementation improves performance status without increasing treatment-related toxicity (93). Furthermore, omega-3 PUFAs mitigate methotrexate-induced hepatotoxicity, maintain liver function, and preserve the balance of the oxidative-antioxidative system (29). Theoretical analysis also suggests that omega-3 PUFAs may protect auditory cells from platinum-related ototoxicity without compromising tumor cell killing or chemotherapy efficacy (94). Collectively, omega-3 PUFAs show promise as adjuvant agents in chemotherapy by enhancing therapeutic response, reducing chemotherapy-related toxicity, and overcoming drug resistance, thereby improving overall treatment outcomes.

Although these findings demonstrate clinical potential, integrating omega-3 PUFAs into adjuvant therapy remains challenging due to several factors, including the determination of optimal dosage, timing of administration, formulation selection, and target population definition. Large-scale, methodologically sound RCTs are still required to further clarify their value and applicability in comprehensive cancer treatment.

### 4.3 Clinical progress

In the PubMed database, we identified 167 clinical trials in this field. After summarizing and analyzing these trials, we found that current research shows the following development trends and focuses: (1) Omega-3 PUFAs provide chemopreventive benefits for high-risk populations by modulating the metabolic and immune microenvironments. Although the preventive effects of omega-3 PUFAs remain unclear in the general population (95, 96), supplementation has been shown to improve the breast tissue microenvironment in individuals with specific risk markers, such as women with increased breast density or atypical hyperplasia. This improvement is reflected in reduced breast density, decreased Ki-67 expression, and improved cytological atypia (97–99). Additionally, a large randomized trial found that daily marine-derived omega-3 PUFA supplementation (1 g/day) did not reduce the overall risk of colorectal precancerous lesions, including conventional adenomas and serrated polyps. However, it may offer potential benefits in subgroups with low baseline omega-3 levels or among African Americans (100). Lung transplant recipients represent a population at extremely high risk for skin cancer. Preliminary trial data suggested that daily high-dose omega-3 PUFAs supplementation may lower skin cancer risk in these patients (101). (2) Omega-3 PUFAs improve the wellbeing and quality of life of patients with cancer-associated cachexia by exerting systemic anti-inflammatory and metabolic regulatory effects (102, 103). They enhance postoperative immune function in tumor patients, reduce infection complications (104, 105), and alleviate chemotherapy-related toxicities (106, 107). (3) Omega-3 PUFAs also may enhance therapeutic response through multimodal combination therapies. For instance, combining omega-3 fatty acids with immune-supporting nutrients such as glutamine and arginine may optimize surgical outcomes (104). Pairing omega-3 PUFAs with vitamin D3 improves the nutritional condition of individuals suffering from breast and colorectal cancer (108, 109). Nutritional support rich in omega-3 PUFAs, micronutrients, and probiotics helps maintain stable body weight in patients with head and neck cancer and cachexia (102). Moreover, several clinical studies have suggested potential benefits of combining omega-3 PUFAs with evening primrose oil (110), celecoxib (111), and a compound supplement containing hydroxytyrosol and curcumin (112). Future research should focus on long-term follow-up studies, establishing dose-response relationship models for different disease stages, and optimizing the formulation ratios of components within multi-component supplements.

Taken together, the identified thematic domains suggest that omega-3 PUFA-related cancer research has evolved along three closely interconnected trajectories. First, mechanistic studies provide the biological rationale for this field by supporting a multi-target model in which omega-3 PUFAs may modulate tumor biology through inflammation resolution, lipid metabolism, oxidative stress, epigenetic regulation, immune modulation, tumor microenvironment remodeling, apoptosis, and ferroptosis. Second, epidemiological studies contribute to defining exposure–risk relationships and potentially susceptible populations; however, the current evidence remains heterogeneous, likely due to differences in cancer type, fatty acid subtype, dietary omega-6/omega-3 balance, exposure assessment methods, biomarker measurements, and population characteristics. Third, clinical studies indicate potential benefits of omega-3 PUFAs in cancer cachexia, perioperative immunonutrition, treatment-related toxicity, and adjunctive therapy. Nevertheless, these findings are not fully consistent, partly because of variations in dosage, formulation, intervention timing, treatment context, sample size, and clinical endpoints. Therefore, future studies should integrate mechanistic biomarkers, standardized exposure assessment, dose–response modeling, patient stratification, and clinically meaningful outcomes to better define the patient populations, intervention conditions, and clinical settings in which omega-3 PUFAs may provide the greatest translational value.

### 4.4 Limitations

This study systematically mapped the current status, research hotspots, and future directions of omega-3 polyunsaturated fatty acids in cancer research; however, several limitations should be acknowledged. First, although WoSCC and Scopus are widely used multidisciplinary databases with broad literature coverage and relatively comprehensive citation information, no bibliometric analysis can fully cover all available databases. Therefore, database coverage bias and the omission of relevant publications are difficult to completely avoid. To mitigate this limitation, WoSCC was used as the primary data source, while Scopus data and PubMed clinical trial records were incorporated as complementary sources to enhance the breadth and robustness of the findings. Second, non-English publications were excluded, which may have led to the omission of relevant contributions from other linguistic contexts. In addition, citation-based indicators may be influenced by publication year, journal visibility, research popularity, and field-specific citation practices; therefore, citation counts should not be interpreted as direct measures of study quality or clinical importance. Finally, bibliometric analysis is primarily designed to reveal publication patterns, citation relationships, and thematic structures, but it cannot directly assess the methodological quality, consistency of findings, or certainty of evidence of individual studies. Therefore, future systematic reviews and meta-analyses are needed to further evaluate the evidence quality and clinical implications of specific applications of omega-3 PUFAs in oncology.

## 5 Conclusion

This bibliometric and visual analysis shows that omega-3 PUFA-related cancer research has progressed from broad descriptive and mechanistic exploration toward a more translational field of nutritional oncology. The current knowledge structure is organized around three interconnected domains: mechanistic studies addressing inflammation, apoptosis, ferroptosis, epigenetic regulation, and treatment synergy; population-based studies examining dietary intake, biomarker status, and cancer risk; and clinical studies focusing on cachexia, perioperative immunonutrition, treatment-related toxicity, and adjunctive therapy. However, the translational value of omega-3 PUFAs remains constrained by heterogeneity in exposure assessment, fatty acid subtype, baseline nutritional status, dosage, formulation, intervention timing, patient characteristics, and clinical endpoints. Future studies should integrate molecular mechanisms, objective biomarkers, dose–response modeling, patient stratification, and clinically meaningful outcomes to better define target populations, optimal formulations and dosages, and appropriate intervention windows.

## Data Availability

Publicly available datasets were analyzed in this study. This data can be found at: Web of Science (https://www.webofscience.com); Scopus (https://www.scopus.com/); Pubmed (https://pubmed.ncbi.nlm.nih.gov/).
