# Supplementary figures and images for: Bibliometric and visual analysis of omega-3 polyunsaturated fatty acids in cancer research (2000–2025)
Source: Front Nutr. 2026 Jul 8;13:1818038. doi: 10.3389/fnut.2026.1818038 (PMC13388407; doi:10.3389/fnut.2026.1818038)

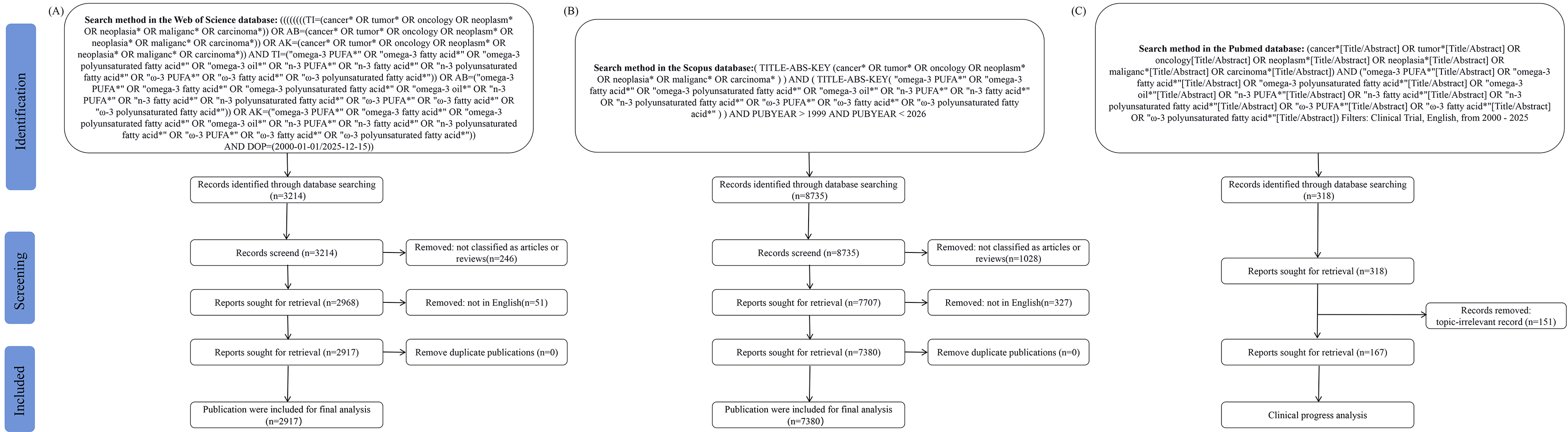

Supplement: Supplementary file 5 [file Image_1.tif]

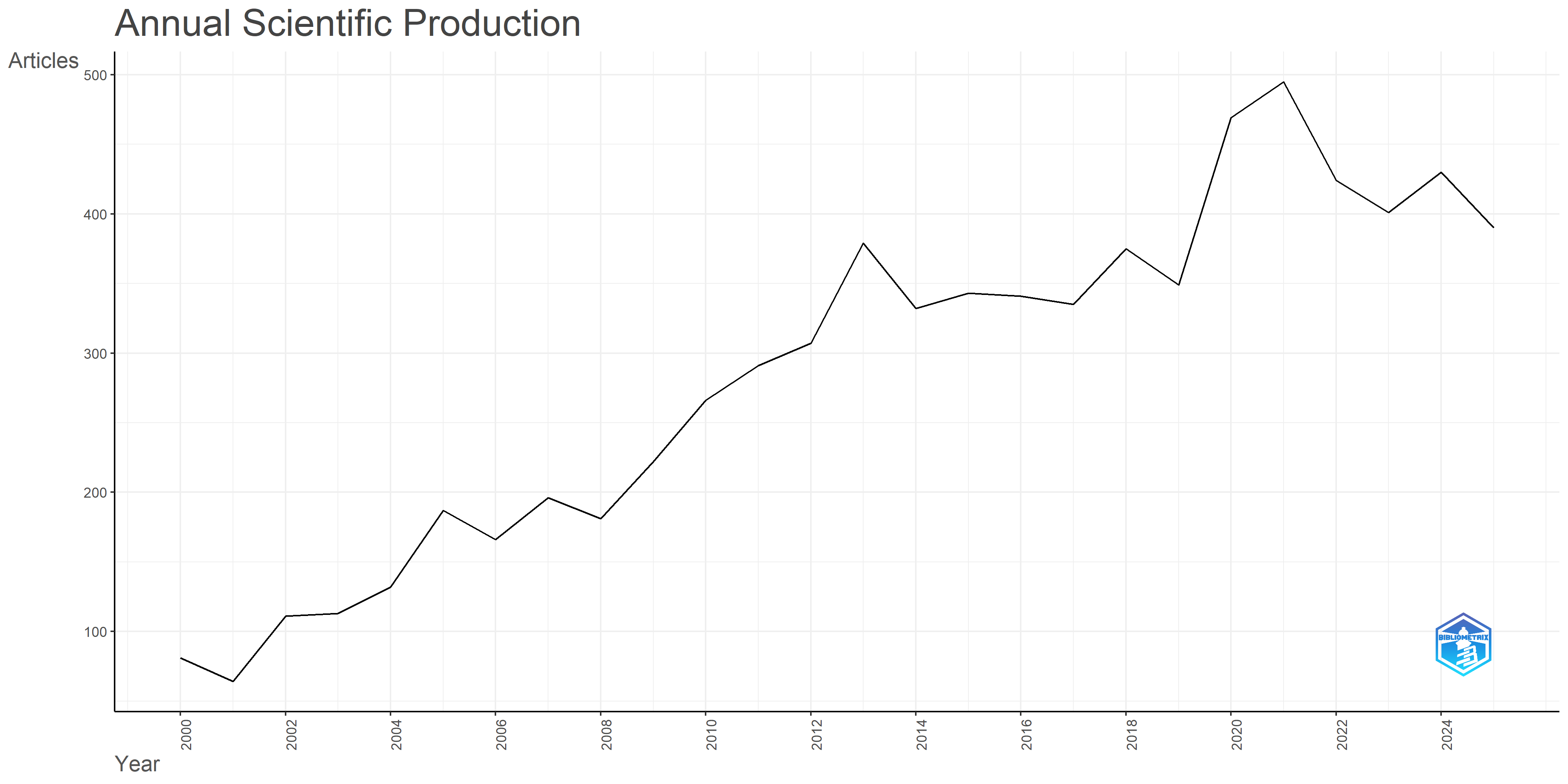

Supplement: Supplementary file 6 [file Image_2.tif]

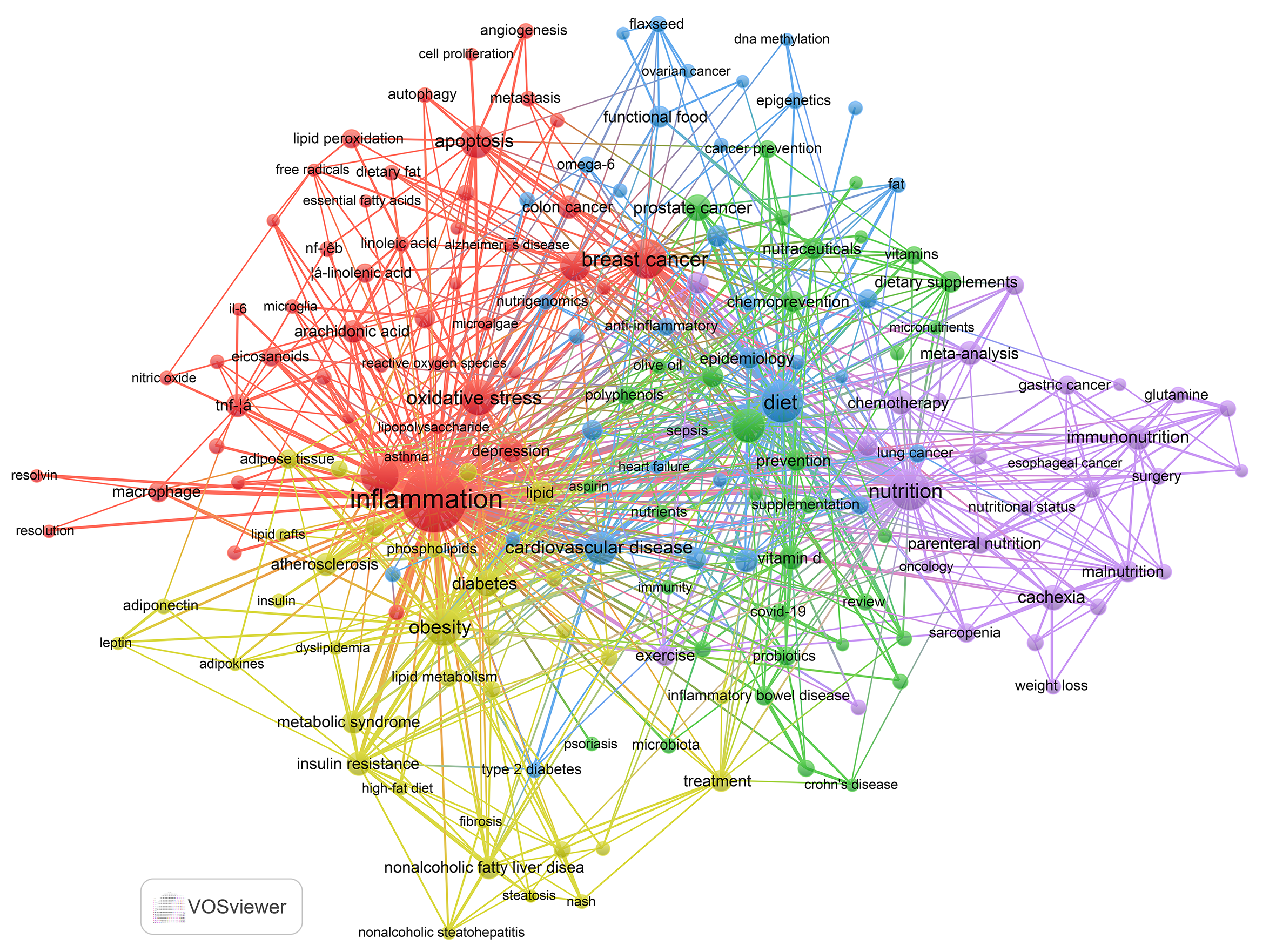

Supplement: Supplementary file 7 [file Image_3.tif]
